# Intake of foods high in saturated fats, vegetarian dietary pattern, and sociodemographic characteristics associated with body weight in Peruvian university students

**DOI:** 10.3389/fnut.2024.1361091

**Published:** 2024-03-20

**Authors:** Jacksaint Saintila, Percy G. Ruiz Mamani, Cristian Ramos-Vera, Antonio Serpa-Barrientos, Susan M. Oblitas-Guerrero, Isabel G. Lizarraga-De-Maguiña, Yaquelin E. Calizaya-Milla

**Affiliations:** ^1^Escuela de Medicina Humana, Universidad Señor de Sipán, Chiclayo, Peru; ^2^Escuela de Enfermería, Universidad Privada San Juan Bautista, Lima, Peru; ^3^Area de Investigación, Universidad Cesar Vallejo (UCV), Lima, Peru; ^4^Departamento de Psicología, Universidad Nacional Mayor de San Marcos, Lima, Peru; ^5^Escuela de Enfermería, Universidad Señor de Sipán, Chiclayo, Peru; ^6^Research Group for Nutrition and Lifestyle, Universidad Peruana Unión, Lima, Peru

**Keywords:** body weight, healthy lifestyle, obesity, risk factors, saturated fats, vegetarians, university students

## Abstract

**Background:**

The prevalence of obesity continues to increase among university students and the general population. Consumption of a diet high in saturated fats could be one of the risk factors.

**Objective:**

The consumption of foods high in saturated fats, the vegetarian diet pattern, and sociodemographic characteristics associated with excess body weight (overweight/obesity) were evaluated in Peruvian university students.

**Methods:**

A cross-sectional study was carried out selecting 5,608 Peruvian university students through no probabilistic convenience sampling. The survey was carried out during the months of February and April 2022. The Chi-square test and binary logistic regression analysis were used to evaluate the association between diet (saturated fats intake and dietary pattern) and sociodemographic factors with excess body weight in a cross-sectional analysis.

**Results:**

It was observed that students who reported high consumption of foods high in saturated fats (OR_B_ = 1.14) and those who had a non-vegetarian dietary pattern (OR_B_ = 2.76) were found to be more likely to have excess body weight. On the contrary, students who reported adherence to the vegetarian diet pattern for more than 5 years were less likely to be overweight or obese (OR_B_ = 0.84). Being ≥26 years of age (OR_B_ = 3.28), living in urban areas (OR_B_ = 1.68) and coastal areas of the country (OR_B_ = 1.17), and enrolled in the engineering faculty (OR_B_ = 1.19), were significantly associated with excess body weight.

**Conclusion:**

The findings of the current study evidenced several factors associated with excess body weight in university students. Therefore, it is necessary to promote and implement healthy lifestyle programs, considering sociodemographic and dietary aspects such as age and dietary intake to control and prevent obesity in university students.

## Introduction

The prevalence of obesity continues to rise among university students and the general population, and although there are several examples of national strategies and policies to address this problem, their effectiveness may vary (1, 2). Since 1980, the prevalence of obesity has doubled, equivalent to almost one third of the world’s population (3). In 2016, the World Health Organization (WHO) published a report stating that 39% of adults over the age of 18 were overweight, while 13% were obese (4). Annual deaths due to obesity amount to approximately 2.8 million (5). In Peru, obesity represents a serious public health problem. According to a report from the Instituto Nacional de Salud, 22.3% of the population over 15 years of age suffers from obesity (6). In particular, the university population is not exempt from this problem; in fact, one study reported that 34.4% of Peruvian university students had a BMI ≥ 25 kg/m^2^ (7). These data were confirmed in another similar study, which reported a higher prevalence of obesity in men than in women (8).

The transition between high school and university is a major change in the lives of university students, which in turn implies a risk of weight gain, especially during the first year of university studies (9). Body weight gain at this critical stage may be due to various factors such as the formation of unhealthy lifestyle habits (10), including low levels of physical activity (11), unhealthy eating habits (12), inadequate sleep habits and patterns (13), as well as alcohol consumption (14). Factors influencing unhealthy lifestyles among university students are multiple, including a sense of independence over their food choices and multiple unhealthy food options, such as those high in saturated fats (15).

Consumption of foods high in saturated fats is undoubtedly one of the main factors that influence the body composition of university students (10). Several studies have reported associations between unhealthy diet practices and obesity in university students. A survey of 300 university students found an association between a high prevalence of fast-food consumption and obesity; in addition, the study showed that the consumption of high-calorie foods is associated with abdominal obesity (16). Other studies report that approximately 30% of high school adolescents and more than 50% of university students consume fast-foods (17, 18), which are rich in saturated fats, trans fats, and cholesterol. In the current study, we did not evaluate fast-food itself; however, most of the foods evaluated, such as hamburgers, french fries, pizza, cakes, and others, are commonly found in fast food restaurants (19).

Prevention of obesity can be achieved by promoting a healthy lifestyle that includes healthy dietary patterns such as vegetarian diets with an emphasis on minimally processed and natural foods such as fruits, vegetables, nuts, whole grains, and seeds and a lower consumption of ultra-processed foods rich in saturated and trans fats (20). Furthermore, other healthy foods, such as fish and fermented or low-fat dairy products, may be beneficial in the prevention of obesity (21). In fact, a study conducted on university students found that those who followed a plant-based diet had lower waist circumference and lower systolic blood pressure (22).

However, dietary patterns that emphasize the consumption of animal products are characterized by high levels of saturated and trans fats (23). Regular meat consumption is associated with alterations in anthropometric parameters and lipid profiles, reflected in increases in body mass index (BMI), waist circumference, and body fat percentage, as well as triglycerides, low-density lipoprotein, and glucose levels (24, 25). These changes contribute to a greater predisposition to cardiometabolic risks and diseases such as obesity, hypertension, diabetes mellitus, and cardiovascular pathologies.

Excess body weight (overweight/obesity) is one of the most important risk factors for noncommunicable diseases such as type 2 diabetes mellitus, cardiovascular disease (CVD), and different types of cancer (26, 27). Moreover, dietary patterns dominated by an intake of saturated fat, trans fats, and cholesterol increase blood cholesterol levels, which, in turn, negatively impact cardiovascular health (26). Considering this reality, the Peruvian government approved the Advertising Warning Manual within the framework of Law No. 30021, the Law for the Promotion of Healthy Eating, whose purpose is to inform the population about the nutritional content of processed and ultra-processed foods to reduce the risk factors associated with obesity and CVD (28). Against this scenario, the national food production industry is required to put a front nutritional warning label on processed foods that are high in sugar, sodium, and saturated fats and eliminate the trans fatty acid content in the products (29).

International organizations such as the American Heart Association recommend that saturated fat intake for apparently healthy adults be limited to less than 7% of total daily calories, trans fat to less than 1% of total daily calories, and cholesterol to less than 300 mg per day (30). In Peru, since March 2021, the Peruvian government requires that no processed and ultra-processed food products such as creams, butter, beef fat (tallow), chicken fat, pork fat (lard), stick margarine and vegetable shortening contain trans fats (28). Substantial changes in student dietary patterns are an important factor in the obesity epidemic and justify the need to evaluate these dietary factors to formulate nutritional intervention programs in this specific population. This study aims to determine the sociodemographic characteristics, dietary pattern, and fat intake associated with excess body weight (overweight/obesity) among Peruvian university students.

## Materials and methods

### Design and participants

**Figure 1:**
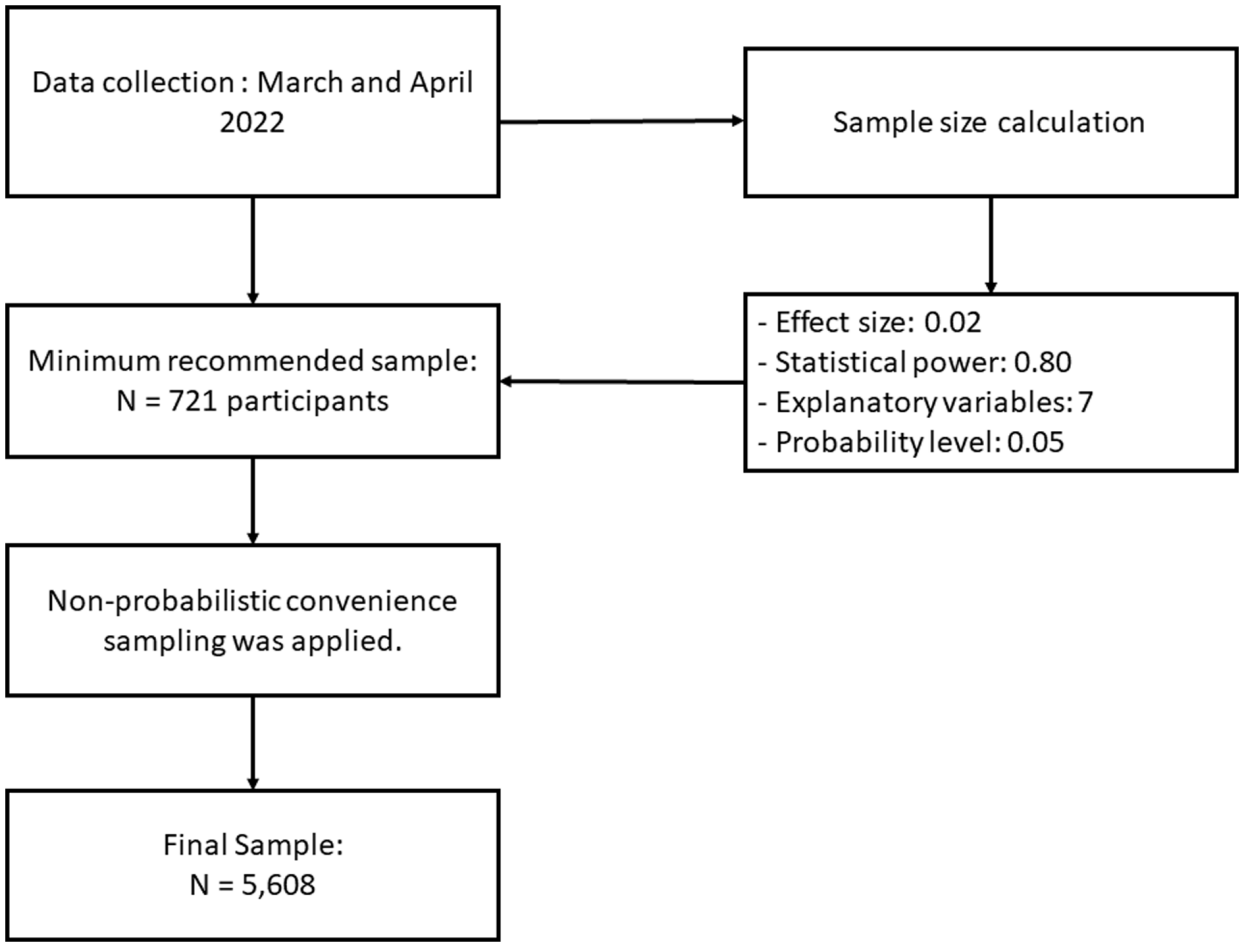
Study design.

The study was conducted based on an online cross-sectional design. Data from students at a private university in Peru were collected during the months of February and April 2022 through non-probabilistic convenience sampling (31–33). The researchers chose to use this sampling because it was the most practical option for obtaining data from respondents (31, 32). The sample size was calculated using Free Statistic Calculators version 4.0 (34). For the multiple regression analysis, an effect size of 0.02, a statistical power of 0.80, seven explanatory variables and a probability level of 0.05 were considered. According to this calculation, a minimum sample of 721 participants was required. However, a total of 5,608 students voluntarily participated in this study, exceeding the calculated sample size (Figure 1). All participants were recruited through an invitation sent via the university’s virtual platform, in which the purposes of the research and their rights as potential participants were briefly described. The participants then gave their informed consent by clicking on the “I wish to participate” option after reading the approved informed consent form on the preliminary page of the questionnaire. Participation was voluntary and did not offer them any incentive to respond to the survey. The questionnaire was online through the university’s virtual survey system. Students under 18 years of age and those who did not correctly answer the questions on the questionnaire were excluded. Before conducting the investigation, the procedures were reviewed and approval was obtained from the Research Ethics Committee of the Universidad Peruana Unión and registered under the number 2022-CEUPeU-0009. Finally, the study was conducted in accordance with the Declaration of Helsinki.

### Variables

#### Intake of foods high in saturated fats

A food frequency questionnaire created and validated in a previous study was used to assess saturated fat intake (35). The instrument was previously used in the Peruvian population (36) and validated in university students (37). The questionnaire consists of 16 items and is measured with scales that determine the following frequencies: 1 time per month or less = 0 point, 2 to 3 times a month = 1 point, 1 to 2 times a week = 2 points, 3 to 4 times a week = 3 points and 5 or more times a week = 4 points. The sum of the scores corresponding to 0–7 points equals a very low fat intake, 8–14 points equals a medium fat intake, 15–22 points equals a high fat intake, and 23 or more points equal a very high fat intake (Appendix A). The robustness of the questionnaire was demonstrated by coefficients greater than 0.90, with an ordinal alpha of 0.94, an omega of 0.94, and an H of 0.95 (37). These results underscore the high reliability and relevance of the instrument to assess saturated fat intake in the target population.

#### Vegetarian dietary pattern

The dietary pattern of the students was self-reported through the following question: *“What type of diet do you follow?”* and classified as non-vegetarian (those who reported consuming red meat and derivatives, poultry, fish, and vegetables), semi-vegetarian (those who consumed red meat, poultry and fish less than once per week and more than once per month) (38, 39), and vegetarian (those who reported consuming milk, eggs, derivatives, and vegetables) (20, 40). In addition, we assessed adherence to the vegetarian dietary pattern using the following single item: *How long have you been a vegetarian?* The response options were (a) < 3 years, (b) 3–5 years, and (c) > 5 years. To determine these categories, we used the existing literature, considering that a significant effect on body weight is observed in people who have followed a vegetarian diet for at least 5 years (41–44).

#### Sociodemographic characteristics

To collect sociodemographic data, a registration form was used as part of the survey. Specific information was collected on sex, age, marital status (married and single), place of origin (coast, jungle, and highlands), place of residence (urban and rural), religious affiliation (Adventist, Baptist, Catholic, and Evangelical), area of study (health sciences, engineering and architecture, human sciences, and business), and parents’ level of education (basic, technical, bachelor’s degree, and postgraduate).

#### BMI

Participants reported their weight and height. BMI was calculated according to the Quetlet index, according to the criteria established by the WHO (45). Likewise, it was classified as follows: (a) underweight, <18.5; (b) normal, 18.5–24.9 kg/m2; (c) overweight, between 25.0–29.9 kg/m2; (d) obesity ≥30 (45).

### Statistical analysis

Statistical analyzes were performed using the IBM SPSS statistical program, version 24 (SPSS Inc., Chicago, IL, USA). The results were reported in tables and text by calculating absolute frequencies and percentages. In addition, an analysis of the association between sociodemographic characteristics, dietary pattern, and fat intake with excess body weight (overweight/obesity) was carried out. The Chi-square test was used. A binary logistic regression model was used for the analysis of the association between factors associated with excess body weight (dependent variable). Independent variables such as age, origin, place of residence, faculty, dietary pattern, intake of foods high in saturated fats and adherence to the vegetarian dietary pattern were included. These variables were included in the model because they had a probability value (*p*-value) less than 0.05 when the bivariate analysis was performed. All analyzes were performed considering a significance level of 5%.

## Results

A total of 5,608 university students decided to participate in the study. 2,990 (53.32%) were men and 2,618 (46.68%) were women. The mean age of the participants was 21.5 ± 3.19 in women and 22 ± 3.60 in men. The description of the sociodemographic characteristics of the participants is shown in Table 1. It was observed that 89.6% of the participants were students between 18 and 25 years of age, of which 91.8% were female (*p* < 0.001). In addition, 94.7% were single and more than half (57.3%) were from the highlands of the country. Similarly, 70.2% lived in urban areas and more than half of the parents of university students had a basic level of education (58.8%). The students were mainly (53.6%) members of the Seventh-day Adventist Church (Seventh-day Adventist Church), with men being more likely to profess that religion than women (54.7% vs. 52.6%, *p* = 0.013). Finally, students of health science are more likely to be women (44.4%), while 46.6% of those in the faculty of engineering and architecture were male (*p* < 0.001).

**Table 1:** Sociodemographic characteristics according to the sex of university students.

	Women		Men		Total			
Variable	*n*	%	*n*	%	*n*	%	*χ*2	*p*
Age (years)								
18–25	2,746	91.8	2,278	87.0	5,024	89.6	34.854	< 0.001*
26–30	177	5.9	247	9.4	424	7.6		
31–36	67	2.2	93	3.6	160	2.9		
Marital status								
Married	154	5.2	143	5.5	297	5.3	0.270	0.603
Single	2,836	94.8	2,475	94.5	5,311	94.7		
Region of origin								
Coast	605	20.2	534	20.4	1,139	20.3	3.926	0.270
Highlands	1702	56.9	1,510	57.7	3,212	57.3		
Jungle	619	20.7	502	19.2	1,121	20.0		
Foreign	64	2.1	72	2.8	136	2.4		
Area of residence								
Rural	915	30.6	755	28.8	1,670	29.8	2.075	0.150
Urban	2075	69.4	1863	71.2	3,938	70.2		
Parents’ level of education							3.982	0.263
Basic	1782	59.6	1,516	57.9	3,298	58.8		
Technical	521	17.4	449	17.2	970	17.3		
Bachelor’s Degree	444	14.8	405	15.5	849	15.1		
Postgraduate	243	8.1	248	9.5	491	8.8		
Religion								
Adventist	1,573	52.6	1,432	54.7	3,005	53.6	10.734	0.013*
Baptist	165	5.5	175	6.7	340	6.1		
Catholic	1,007	33.7	784	29.9	1791	31.9		
Evangelical	245	8.2	227	8.7	472	8.4		
Study area								
Health Sciences	1,328	44.4	467	17.8	1795	32.0	714.817	< 0.001*
Humanities	303	10.1	262	10.0	565	10.1		
Engineering and Architecture	503	16.8	1,219	46.6	1722	30.7		
Business	856	28.6	670	25.6	1,526	27.2		

*p < 0.05 (significant).

Table 2 shows the dietary pattern, the intake of saturated fats and the BMI according to the sex of the participants. The intake of saturated fats was higher in men than in women (43.3% vs. 30.9%, *p* < 0.001). On the other hand, vegetarian diets were represented by 32.2% of men compared to women (24.7%), *p* < 0.001. The percentage of excess body weight was slightly higher in men (35.8%) than in women (34.7%), however, there were no statistically significant differences (*p* = 0.410).

**Table 2:** Dietary pattern, saturated fat intake, and BMI according to the sex of the participants.

Variable	Women		Men		Total			
	*n*	%	*n*	%	*n*	%	χ2	*p*
Dietary pattern							44.642	< 0.001*
Vegetarian	739	24.7	845	32.2	1,584	28.2		
Semi-vegetarian	1,238	41.4	925	35.3	2,163	38.6		
No-vegetarian	1,013	33.9	848	32.4	1861	33.2		
Saturated fat intake							92.542	< 0.001*
Low intake	2067	69.1	1,485	56.7	3,552	63.3		
High intake	923	30.9	1,133	43.3	2056	36.7		
BMI							2.883	0.410
Underweight	90	3	92	3.5	182	3.2		
Normal	1862	62.3	1,587	60.6	3,449	61.5		
Overweight	891	29.8	794	30.3	1,685	30		
Obesity	147	4.9	145	5.5	292	5.2		

*p < 0.05 (significant).

Table 3 shows that students who were ≥ 26 were more likely to report excess body weight (*p* < 0.001). Similarly, those who reported high saturated fat intake, who lived in the coastal region of the country and urban areas, those enrolled in engineering faculty, and those who had a non-vegetarian dietary pattern were more likely to have excess body weight (*p* < 0.05). On the other hand, students who adhered to the vegetarian dietary pattern for more than 5 years reported adequate weight (*p* < 0.001).

**Table 3:** Analysis of sociodemographic characteristics, dietary pattern, and intake of foods high in saturated fats according to the body weight status of the participants.

	With EBW		No EBW		Total			
	*n*	%	*n*	%	*N*	%	*X* ^2^	*p*
Age (years)							146.301	< 0.001*
18–25	1,641	83.0	3,383	93.2	5,024	89.6		
26–30	233	11.8	191	5.3	424	7.6		
31–36	103	5.2	57	1.6	160	2.9		
Sex							0.811	0.368
Female	1,038	52.5	1952	53.8	2,990	53.3		
Male	939	47.5	1,679	46.2	2,618	46.7		
Region of origin							73.920	< 0.001*
Coast	508	25.7	631	17.4	1,139	20.3		
Highlands	1,012	51.2	2,200	60.6	3,212	57.3		
Jungle	391	19.8	730	20.1	1,121	20.0		
Foreign	66	3.3	70	1.9	136	2.4		
Place of residence							28.101	< 0.001*
Rural	502	25.4	1,168	32.2	1,670	29.8		
Urban	1,475	74.6	2,463	67.8	3,938	70.2		
Religion							17.773	< 0.001*
Adventist	1,047	53.0	1958	53.9	3,005	53.6		
Baptist	155	7.8	185	5.1	340	6.1		
Catholic	606	30.7	1,185	32.6	1791	31.9		
Evangelical	169	8.5	303	8.3	472	8.4		
Study area							12.147	0.007*
Health Sciences	586	29.6	1,209	33.3	1795	32.0		
Humanities	219	11.1	346	9.5	565	10.1		
Engineering and Architecture	644	32.6	1,078	29.7	1722	30.7		
Business	528	26.7	998	27.5	1,526	27.2		
Dietary pattern							276.470	< 0.001*
Vegetarian	457	20.0	1,127	34.0	1,584	28.2		
Semi-vegetarian	899	39.2	1,264	38.1	2,163	38.6		
No-vegetarian	935	40.8	926	27.9	1861	33.6		
Vegetarian dietary pattern adherence							14.290	< 0.001*
< 3 years	541	32.0	211	10.3	752	20.1		
3–5 years	615	36.4	250	12.2	865	23.1		
> 5 years	534	31.6	1,596	77.5	2,130	56.8		
Saturated fats intake							11.393	< 0.001*
Low intake	1,194	60.4	2,358	64.9	3,552	63.3		
High intake	783	39.6	1,273	35.1	2056	36.7		

*p < 0.05 (significant). EBW = excess body weight (overweight/obesity).

The following factors were included in the multivariate analysis: intake of foods high in saturated fats (0 = low intake; 1 = high intake), dietary pattern (0 = vegetarian; 1 = no vegetarian), adherence to the vegetarian dietary pattern (0 = < 5 years; 1 = > 5 years), age (years) (0 = < 26; 1 = ≥ 26), place of origin (0 = other regions; 1 = coast), place of residence (0 = rural; 1 = urban), and faculty (0 = other faculties; 1 = engineering). It was found that high intake of foods high in saturated fats (*β* = 0.13; OR_B_ = 1.14; *p* = 0.02), non-vegetarian dietary pattern (*β* = 1.01; OR_B_ = 2.76; *p* < 0.001), being ≥26 years old (*β* = 1.18; OR_B_ = 3.28; *p* < 0.001), living in urban (*β* = 0.16; OR_B_ = 1.68; *p* = 0.015), and coastal areas of the country (*β* = 0.52; OR_B_ = 1.17; *p* < 0.001), and enrolled in the faculty of engineering (*β* = 0.17; OR_B_ = 1.19; *p* = 0.006), were significantly associated with excess body weight. However, adherence to the vegetarian diet pattern for more than 5 years (*β* = −0.17; OR_B_ = 0.842; *p* = 0.005) was associated with a lower likelihood of having excess body weight (Table 4).

**Table 4:** Multivariable association analysis of factors associated with excess body weight (overweight/obesity) in university students.

				95% CI	
Model	*B*	OR_B_	*p*	Lower	Upper
Saturated fat intake (0 = Low intake; 1 = High intake)	0.13	1.14	0.025	1.01	1.29
Vegetarian dietary pattern (0 = Vegetarian; 1 = Non-vegetarian)	1.01	2.76	< 0.001	2.44	3.13
Adherence to dietary pattern (0 = < 5 years; 1 = > 5 years)	−0.17	0.84	0.005	0.74	0.95
Age (years) (0 = < 26; 1 = ≥ 26)	1.18	3.28	< 0.001	2.73	3.94
Place of residence (0 = Rural; 1 = Urban)	0.16	1.17	0.015	1.03	1.33
Faculty (0 = Other faculties; 1 = Engineering)	0.17	1.19	0.006	1.05	1.35
Place origin (0 = Other regions; 1 = Coast)	0.52	1.68	< 0.001	1.47	1.92

B = Beta coefficient, *p* = probability, OR_B_ = multivariate risk, 95% CI = 95% confidence interval.

## Discussion

Prevalence of obesity continues to increase among university students and the population in general (7, 8, 46). The consumption of foods high in saturated fats constitutes one of the main risk factors influencing obesity in university students (10, 19, 36). Practicing a healthy lifestyle that includes a balanced diet based on the consumption of minimally processed plant foods can prevent negative changes in body composition (22). This study aimed to evaluate the association between saturated fat intake, vegetarian status, sociodemographic characteristics, and excess body weight among Peruvian university students. The relevant findings of this study were: bivariate analysis of factors associated with excess body weight showed a high intake of saturated fat, a non-vegetarian dietary pattern, ≥ 26 years, living in the coastal region and urban areas of the country, and enrolled in the engineering faculty to be statistically significantly associated with excess body weight. After performing a logistic regression analysis, these variables were significantly associated with excess body weight in university students. In contrast, we have found that adhering to a vegetarian dietary pattern for more than 12 months is associated with less likelihood of excess body weight (overweight/obesity) compared to adhering for less than 5 years.

## Influence of saturated fats intake

Substantial consumption of foods high in saturated fats among university students represents an elevated risk of long-term weight gain and the development of other health conditions such as metabolic syndrome, insulin resistance, and type 2 diabetes (47). In the current study, high consumption of foods high in saturated fats is associated with excess body weight. These results are in line with the findings of a study in university students in which the consumption of high-fat foods was associated with a poorer health status according to BMI scores (48). In addition, it is important to mention that the type of fat influences obesity. In fact, a study reported that participants who used butter or butter-oil-fat spreads were less likely to have a body weight problem than those who did not apply any on bread, while sausage consumption in women increased the risk (49). It is worth mentioning that the consumption of saturated fats and their relationship with body weight gain remains a controversial issue. For example, there is evidence that fat intake between 18 and 40% of energy intake has little or no effect on adiposity (50), while other studies claim that fat intake energy increases the risk of obesity (51). However, fat intake alone is not the only factor affecting body weight; it is a grouping of different factors such as a low level of physical activity, poor diet, smoking, and heredity, all of which contribute to weight gain (52).

On the other hand, the results of the study indicated that fat intake was more prevalent in men; moreover, they had a slight excess of body weight compared to women. Our findings are consistent with the results of a similar study conducted in US university students that concluded that saturated fat and cholesterol intake was lower in female students (23.5 g/day) than in male students (28.9 g/day) (10). Similarly, findings from an Iranian study of 184 university students showed that female students had a significantly lower intake of saturated fat and a higher intake of monounsaturated fat compared to male students (53). In addition, this same study reported that women perceived themselves as healthy based on body weight (53). These findings were not surprising, considering that women are generally more likely to opt for a healthy diet because they are more concerned about food and body weight, especially during their university years (54). Another study conducted in a group of university students reported that, although female students were stressed, they reported healthy eating habits compared to males, who had a higher level of excess body weight and little interest in dietary advice and health improvement activities (55). Additionally, the study (55) reported that women were more willing to initiate changes in their eating habits than their male counterparts.

## Vegetarian dietary pattern

Vegetarian (vegan, lacto-ovo-vegetarian, and pesco-vegetarian) and semi-vegetarian (those consuming red meat, poultry, or fish no more than once a week) diets were more frequent among students without excess body weight. Furthermore, according to binary logistic regression analysis, we found that the non-vegetarian dietary pattern was associated with excess body weight. These findings are supported by the results of previous studies conducted in vegetarians and non-vegetarians in Peru (24, 56), in which vegetarians were found to have a lower BMI compared to non-vegetarians. In addition, Olfert et al. conducted a study of 1,078 vegetarian and non-vegetarian university students and found that those who reported opting for a plant-based diet had a lower BMI compared to meat eaters (22). In addition, the results of two studies carried out in Taiwanese vegetarians (57) and Brazilians (58), found that the BMI of non-vegetarians was >27.0 kg/m2 and > 24.9 kg/m2, respectively, relative to their vegetarian counterparts. These results could be explained by the fact that plant-based diets contain a lower caloric density, due to the presence of a higher content of dietary fiber and bioactive elements (59). The health benefits of vegetarian diets in BMI are well documented in the scientific literature. Current studies have suggested that vegetarian diets, particularly vegan diets, are essentially antiobesogenic compared to diets that include meat and derived products (60). There are several studies that suggest that vegetarianism is significantly associated with lower BMI, total cholesterol concentration, LDL cholesterol, glucose levels, systolic blood pressure, diastolic blood pressure (24, 56, 61, 62), and a lower risk of all-cause mortality (63).

Diets based on animal food consumption are characterized by high caloric density, due to excess saturated and trans fats and added sugars (23). Regular meat intake can have a negative impact on the anthropometric and lipid profile, leading to elevated BMI, waist circumference and fat percentage, higher triglycerides, low-density lipoproteins, and glucose concentrations, which, in turn, increase cardiometabolic risk factors, obesity, hypertension, diabetes mellitus, and cardiovascular disease (24, 25). Therefore, it is important to encourage the adoption of a plant-based diet in university students to reduce risk factors for obesity and other diet-associated diseases based on the consumption of meat and meat products.

The present study has shed light on the significant influence that the duration of adherence to a dietary pattern has on body weight in university students. A striking finding of the study is the association between the prolonged adoption of a vegetarian diet and the reduced prevalence of overweight or obesity. The data suggest that those students who have maintained a vegetarian diet for more than 5 years are less likely to have weight problems than their non-vegetarian peers. These results support the findings of previous studies that vegetarians with less than 5 years of dietary follow-up do not have significant differences in BMI compared to individuals consuming omnivorous diets (41, 64). This underscores that the benefits of maintaining a healthy weight are only realized when the vegetarian diet is followed consistently and regularly over time (42–44, 64). In this sense, the temporary or recent adoption of such a diet does not appear to confer the same protection against overweight or obesity.

Vegetarian diets (pesco-vegetarian, lacto-vegetarian, ovo-vegetarian, lacto-ovo-vegetarian, and vegan) showed a significantly higher proportion among men. However, previous studies conducted on vegetarians and non-vegetarians in the general Peruvian population reported that there was a higher percentage of female vegetarians compared to male vegetarians (24, 56). Similarly, studies conducted in another context showed that most women tend to opt for plant-based diets (22). However, in order to contextualize the fact that there is a higher proportion of male vegetarians in the current study, it is important to consider several sociocultural factors that could influence the choice of a vegetarian diet. For example, an aspect to consider is the availability and accessibility of vegetarian options in the environment, as the university where the study took place is part of the SDA Church, a religious denomination whose health philosophy focuses on the consumption of plant-based foods (65). As vegetarian options become more varied and accessible in certain settings, more men may be inclined to try this type of diet.

It is important to note that the fact that there was a higher proportion of vegetarian men than women does not guarantee that fewer men ate foods high in saturated fats. Therefore, it is important to recognize that a vegetarian diet does not automatically equate to a healthy diet. The nutritional quality of a vegetarian diet can vary widely depending on specific dietary choices. Some foods rich in saturated fat, such as chips, cakes, butter, mayonnaise, cheese, whole milk, and cookies, were included in our analysis. It is possible that the men in our sample had a higher preference for these foods compared to the women. This phenomenon highlights the diversity within vegetarian diets and how they can include less healthy choices that are low in certain nutrients (vitamin B12, iron, zinc, and calcium) and/or rich in highly processed and refined foods that contribute to saturated fat consumption (66). This observation is consistent with previous studies that have documented variability in the quality of vegetarian diets (67). Future analyses could further explore how differences in food choices within vegetarian diets affect the overall nutritional profile and long-term health. Our findings underscore the importance of considering not only diet categorization, but also diet quality and nutrient composition when assessing health outcomes.

## Sociodemographic characteristics

Another important finding to mention is that students ≥26 years were more likely to have excess body weight. Furthermore, the findings of a cross-sectional study of 2,285 Peruvian university students found that those aged ≥27 years were more likely to be overweight/obese (46). Similarly, a study conducted on 4,201 university students found that the mean BMI of the participants increased with age (68). On the other hand, studies conducted in the general population showed that increasing age was a risk factor for obesity (69). One study revealed that older participants had almost twice the risk of being obese (70). As age increases, due to hormonal and metabolic changes (71), there is an increased risk of obesity. Therefore, permanent and conscious control of food intake and regular physical activity practice is important to prevent weight gain.

Additionally, living in the coastal region and urban areas is associated with excess body weight. The coast of Peru has the highest concentration of urban areas. These cities are home to the people most affected by the presence of overweight and obesity and 1 in 4 people are obese (72). One of the possible justifications for this problem is due to the fact that Peruvian society is experiencing transitional phenomena, characterized by high levels of urbanization, modernization, education, income, access to health facilities, basic sanitation, aspects of technology, among others, which are occurring at an accelerated pace and, as a result, some lifestyle behaviors, such as eating habits, physical activity, and rest, were affected (73). University students living in urban areas could be exposed to significant changes in dietary patterns, which, in turn, could negatively impact body composition (74). The rhythm of life in the city replaces traditional dietary activities, characterized by fishing and agriculture, giving rise to a consumption pattern differentiated by a higher intake of ultra-processed foods (75). However, while previous findings suggest that living in rural areas increases the risk of being overweight, our results contradict this trend by showing that students living in urban areas have a higher prevalence of overweight and obesity (69). Therefore, future research should focus on the development of lifestyle-based interventions for university students, considering the geographic areas of residence to prevent excess body weight.

Finally, we found that students enrolled in the Faculty of Engineering were more likely to be overweight than students in other faculties. This finding is consistent with the existing literature suggesting similar patterns in comparable populations (46, 76). These results may be due to the academic load and lifestyle habits associated with time-intensive fields of study, such as engineering, which can lead to limited physical activity and unhealthy eating, increasing the risk of being overweight (77). The sedentary nature of prolonged study and the tendency to skip meals or resort to fast food options due to time constraints and deadlines have also been cited as contributing to obesity in this student population (78). This study adds to the growing body of literature identifying engineering students as a risk group for overweight and obesity. It reflects the importance of a multidisciplinary approach that involves both educators and health professionals in developing strategies to promote healthy habits among this student population.

### Strengths and limitations

This study considered a large sample size (more than 6,000 university students). However, the results of our study should be interpreted considering certain limitations. First, it is a non-randomized sampling of the population studied. Second, although the question assessing the dietary pattern provided a definition of the subgroups of vegetarian diets, however, this variable was self-reported and the information provided may not be accurate. Therefore, future studies should consider the dietary intake to assess the dietary pattern of the participants and not rely on a self-reported pattern. Third, although we have classified the diet into three categories (vegetarian, semi-vegetarian and non-vegetarian), however, we do not know the exact proportion of vegetarian subgroups (pesco-vegetarians, lacto-ovo-vegetarians, and vegans). Finally, although the university where the study was conducted has campuses in three regions of Peru, the findings cannot be generalized to the entire Peruvian population of university students because it is a sampling technique based on a single university.

## Conclusion

This cross-sectional study associated the intake of foods high in saturated fats, vegetarian dietary pattern, and sociodemographic characteristics with body weight in university students. It was found that students who reported a high intake of foods high in saturated fat and those with a non-vegetarian dietary pattern were more likely to be overweight. On the contrary, students who reported adherence to the vegetarian diet pattern for more than 12 months were less likely to be overweight or obese. Furthermore, it was found that being ≥26 years old, living in urban areas and coastal areas of the country, and enrolled in the engineering faculty were significantly associated with excess body weight. Furthermore, men were found to have the highest representation among those following vegetarian diets. Fat intake was more prevalent in men; in addition, they were slightly overweight compared to women. Therefore, it is necessary to promote and implement healthy lifestyle programs, considering dietary factors such as saturated fat intake and sociodemographic aspects such as age, place of residence for the control and prevention of obesity in university students.

## Data availability statement

The raw data supporting the conclusions of this article will be made available by the authors, without undue reservation.

## Ethics statement

The studies involving humans were approved by Research Ethics Committee of the Faculty of Health Sciences of Universidad Peruana Unión: approval number: 2022-CEUPeU-0009. The studies were conducted in accordance with the local legislation and institutional requirements. The participants provided their written informed consent to participate in this study.

## Author contributions

JS: Conceptualization, Project administration, Visualization, Writing – original draft, Writing – review & editing. PR: Data curation, Validation, Writing – review & editing. CR-V: Data curation, Visualization, Writing – review & editing. AS-B: Investigation, Supervision, Visualization, Writing – review & editing. SO-G: Investigation, Supervision, Visualization, Writing – review & editing. IL: Formal analysis, Methodology, Writing – review & editing. YC-M: Conceptualization, Project administration, Visualization, Writing – original draft, Writing – review & editing.
