# Supplementary material for: Intake of foods high in saturated fats, vegetarian dietary pattern, and sociodemographic characteristics associated with body weight in Peruvian university students
Source: Front Nutr. 2024 Mar 20;11:1361091. doi: 10.3389/fnut.2024.1361091 (PMC10987770; doi:10.3389/fnut.2024.1361091)
Supplement: Supplementary file 1 [file Data_Sheet_1.pdf]

Appendix A

Block Fat Screener (BFS-E)- Spanish version

Cribado de grasas de Block

0 = 1 vez al mes o menos/**1 month or less**
1 = de 2 a 3 veces al mes/**2-3 times/month**
2 = 1 a 2 veces a la semana/**1-2 times/week**
3 = 3 a 4 veces a la semana/**3-4 times/week**
4 = 5 a más veces a la semana/**5 or more times/week**

¿Aproximadamente con qué frecuencia consume los siguientes alimentos? / **Approximately how often do you eat the following foods?**					
Ítems/**Items**	**0**	**1**	**2**	**3**	**4**
1. Hamburguesas, carne molida, o tacos/ **Hamburgers, ground beef, meat burritos, tacos**					
2. Carne de res o cerdo, como filetes, asados, costillas o en bocadillos/ **Beef or pork, such as steaks, roasts, ribs, or in sandwiches**					
3. Pollo frito/ **Fried chicken**					
4. Hot dogs o salchicha/ **Hot dogs, or Polish or Italian sausage**					
5. Embutidos, fiambres, o jamón (no bajo en grasas)/ **Cold cuts, lunch meats, ham (not low-fat)**					
6. Tocino o salchicha en el desayuno/ **Bacon or breakfast sausage**					
7. Aderezos para ensaladas (no bajos en grasa)/ **Salad dressings (not low-fat)**					
8. Margarina, mantequilla o mayonesa para cocinar y utilizar sobre pan o papas/ **Margarine, butter or mayo on bread or potatoes**					
9. Huevos (solo claras de huevo)/ **Margarine, butter, or oil for frying eggs (not Egg Beaters or just egg whites)**					
10. Pizza/ **Pizza**					
11. Queso, o queso para untar (no bajo en grasa)/ **Cheese, cheese spread (not low-fat)**					
12. Leche entera/ **Whole milk**					
13. Papas fritas/ **French fries, fried potatoes**					
14. Chips de maíz, papas fritas paquete, palomitas de maíz, o galletas saladas/ **Corn chips, potato chips, popcorn, crackers**					
15. ¿Donas, pasteles, o galletas (no bajas en grasa)/ **Doughnuts, pastries, cake, cookies (not low-fat)**					
16. Helado que no tenga grasa (helados de hierro, de pula de frutas, entre otros)/ **Ice cream (not sherbet or non-fat)**					

Escalas de evaluación**/Scales of Assessment**

Carecterísticas **/Characteristics**	Nivel**/ Level**	Intervalo**/Interval**
General	Bajo/ **Low**	0–6
	Medio/ **Medium**	7–17
	Alto/ **High**	18–64
Femenino/**Female**		
	Bajo/ **Low**	0–6
	Medio/ **Medium**	7–15
	Alto/ **High**	16–64
Masculino/**Male**		

	Bajo/ **Low**	0–7
	Medio/ **Medium**	8–19
	Alto/ **High**	20–64
